# Long-term outcome of posterior fossa medulloblastoma in patients surviving more than 20 years following primary treatment in childhood

**DOI:** 10.1038/s41598-020-66328-8

**Published:** 2020-06-10

**Authors:** Radek Frič, Bernt Johan Due-Tønnessen, Tryggve Lundar, Arild Egge, Bård Kronen Krossnes, Paulina Due-Tønnessen, Einar Stensvold, Petter Brandal

**Affiliations:** 1 Department of Neurosurgery, Oslo University Hospital, Oslo, Norway; 2 Department of Pathology, Oslo University Hospital, Oslo, Norway; 3 Department of Radiology and Nuclear Medicine, Oslo University Hospital, Oslo, Norway; 4 Department of Pediatrics, Oslo University Hospital, Oslo, Norway; 5 Department of Oncology, Oslo University Hospital, Oslo, Norway; 6 Faculty of Medicine, University of Oslo, Oslo, Norway

**Keywords:** Cancer, Oncology, Surgical oncology

## Abstract

The aim of the study was to analyze the long-term outcome (>20 years) after treatment of posterior fossa medulloblastoma (MB) in childhood. We analyzed data from patients treated for posterior fossa MB between 1974 (introduction of the first international treatment protocol in Norway) and 1987 (when use of radiotherapy was abandoned in children under 4 years of age). Out of 47 children, 24 survived >20 years. At the time of analysis, 16 patients (median age 41 years, range 32–52) were alive (median follow-up 34 years, range 30–42), while 8 patients died 22–41 years (median 31 years) after primary treatment: one late death (after 22 years) was due to tumor recurrence whilst other 7 deaths (after 23 to 41 years) were related to the detrimental effects of the treatment (secondary tumors, stroke, severe epilepsy and depression). Observed 20- and 30-year survival rates were 51% and 44%, respectively. Despite successful treatment of MB in childhood and satisfactory tumor control during the first 20 years following primary treatment, our data indicates that even long-term survivors may die from tumor recurrence. However, the main factors causing late mortality and morbidity in long-term survivors seem to be the complications related to radiotherapy given in childhood.

## Introduction

Treatment of children with brain tumors has improved markedly during the last 50 years. Implementation of microsurgery, modern anesthesia and postoperative care along with improved radiological imaging, particularly computed tomography (CT) and magnetic resonance imaging (MRI), have facilitated both staging, surgical resection and follow-up. While advances in the neurosurgical treatment can be documented by reduced perioperative and early postoperative mortality and morbidity^1^, 5- and 10-year survival rates for different neoplastic entities are considered a standard measure of outcome and quality of treatment for the neoplastic disease itself^2,3^, along with increasing focus on survivors‘ quality of life^4–6^ and physical functioning^7^.

It was well recognized already in the 1950’s and -60’s that there were different prognoses for children with various types of posterior fossa tumors^8^. Yet, first after the introduction of systematic use of postoperative radiotherapy and the first international treatment protocol for posterior fossa medulloblastoma (MB) and primitive neuroectodermal tumor (PNET) in Norway in 1974 including adjuvant chemotherapy^9^, the survival rates improved dramatically from close to zero to more than 50%^1^. With the later recognition of the detrimental effects of radiotherapy, particularly when given to small children^6,8,10^, such treatment was thereafter avoided in children under the age of 4 years. In recent years and whenever available, proton radiotherapy is often preferred over photon radiotherapy in pediatric patients with medulloblastoma, because better radiation dose sparing of normal tissue is supposed to lead to lower toxicity^11^.

Having gathered data from 40 years of treatment of pediatric MB and supratentorial PNET^12^, we recognized that there was a substantial subgroup of long-term survivors who still continued to struggle with the late consequences of their life-saving treatment in childhood. Furthermore, we saw patients in whom late recurrences of their neoplastic disease occurred. As these ultra-late aspects have not been sufficiently reflected in the literature^13^, we wish to summarize the experience from this particular subgroup of patients in the present study.

## Methods

The background for this analysis was the patient material included in the retrospective observational study recently published by our group^12^. Here, all consecutive patients younger than 20 years at the time of primary diagnosis were enrolled. All of these were treated at Oslo University Hospital between January 1974 and December 2013 for MB or supratentorial PNET, confirmed after histopathological review according to the World Health Organization (WHO) 2007 classification criteria. This particular study was approved by the Regional Health Ethics Committee South-East (reference number 2013/1859). All methods were performed in accordance with the relevant guidelines and regulations. As the study was designed as a retrospective observational study, the Committee waived the need for informed consent. In 1974, Norwegian patients became included in the first international trial on the use of adjuvant chemotherapy in children with medulloblastoma (International Society of Pediatric Oncology [SIOP] I)^9^. From 1974 until 1987, all children were given craniospinal radiotherapy postoperatively, regardless of age. Adjuvant chemotherapy was administered according to treatment protocols for the given years.

The present analysis focuses on the subgroup of patients with posterior fossa MB who were treated at our institution from 1974 and to the end of 1987, and who survived the first 20 years following primary treatment. A systematic review of medical records was performed, with date for latest follow-up in December 2017. The functional status at this point of time was assessed using Barthel Index that scores the patients’ degree of independence (from 0 to 100) based on their ability to perform ten major activities of daily living (feeding, bathing, grooming, dressing, bowel and bladder control, toilet use, transfers, mobility and stairs)^14^.

Statistical analysis was performed using Statistical Package for the Social Sciences (SPSS™) software, version 23 (IBM Corporation, Armonk, NY, USA).

## Results

Out of 47 children primarily treated for their posterior fossa MB between 1974 and 1987, 21 died within the first five years, including one perioperative death, one death four days after surgery, and one patient who died after six months due to acute leukoencephalopathy following radiotherapy. Thus, observed 5-year survival for the whole cohort was 55%. Another two patients died due to the tumor recurrence 5.1 and 9.9 years after diagnosis, respectively.

**Figure 1:**
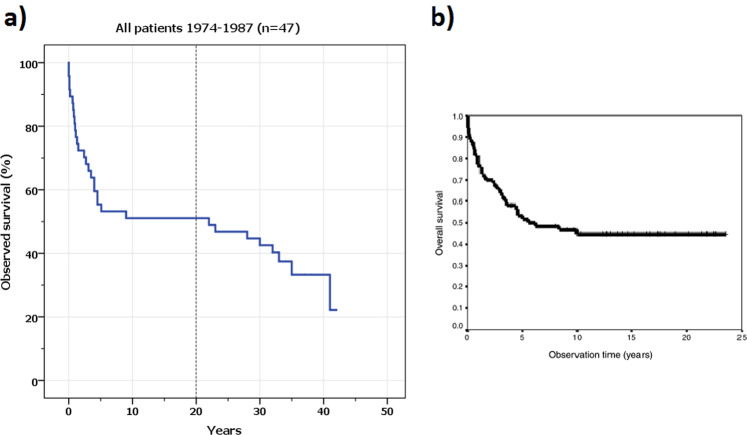
(**a**) Kaplan-Meier curve presenting *observed survival* for children treated for posterior fossa medulloblastoma between 1974 and 1987 (n = 47). Patients included in the present study (n = 24), i.e. surviving >20 years, are those right to the dotted line on the abscissa. **(b)** Kaplan-Meier curve from the publication of Helseth *et al*.^1^ where also some patients from the present study were included, although at that point of time with a mean observation time of only 13.5 years. The decline in survival after 20 years of observation could therefore not be anticipated at the time of this previous publication.

The remaining 24 patients (median age at presentation 7 years, range 0–17; male/female ratio 1:1) survived more than 20 years from primary diagnosis. Hence, the observed 20-year survival in this group was 51% and the median estimated survival for the whole group was 22 years (Fig. 1a). No patient was lost to follow-up.

At the time of analysis (December 2017), 16 patients (median age 41 years, range 32–52, male/female ratio 1:1) from this group were still alive (median follow-up 34 years, range 30–42), while eight patients were deceased from 22 to 41 years (median 31 years) after primary treatment. Out of these, there was one late death after 22 years due to recurrence of the primary disease whilst the other seven cases of late deaths (after 23 to 41 years) appeared to be related to the effect of treatment as further detailed in Table 1.

**Table 1:** Patients surviving >20 years following primary treatment for posterior fossa MB in childhood.

Pt	Diagnosis	Sex/age^a^(years)	Alive	Dead	Follow-up (years)	Education	BI	Marital status	Offspring	Age^b^ (years)	Short clinical summary
1	1974	M/10	No	DOT	32	n/a	0	n/a	n/a	—	multiple meningiomas, **thyroid carcinoma**
2	1975	M/2	**Yes**	—	42	secondary	100	single	no	45	full-time work until 2012, major stroke
3	1975	F/0	No	DOT	41	primary	0	single	no	—	reduced IQ, meningioma, BCC, **asphyxia**^**c**^
4	1975	F/10	No	DOT	35	high	0	n/a	n/a	—	full-time work until 2005, **severe progressive dementia**
5	1976	M/11	No	DOT	23	secondary	0	n/a	n/a	—	**suicide**
6	1976	M/10	**Yes**	—	41	secondary	100	married	2 children	51	part-time work, meningioma, aortic valve insufficiency
7	1978	M/0	No	DOT	30	primary	0	single	no	—	heart valve damage, shunt failure, progressive dementia
8	1979	M/11	**Yes**	—	38	high	100	married	1 child	50	full-time work, reduced hearing
9	1980	F/17	No	DOD	22	secondary	0	married	2 children	—	local tumor **recurrence**
10	1980	M/9	**Yes**	—	37	secondary	100	single	no	46	working until 2006
11	1980	M/3	No	DOT	28	n/a	0	n/a	n/a	—	severe epilepsy leading to fatal **head injury**
12	1980	F/12	**Yes**	—	37	high	100	married	1 child	49	part-time work, petroclival meningioma
13	1981	F/3	No	DOT	33	primary	0	single	no	—	aorta valve insuffiency, dementia, severe depression, **shunt failure**^**d**^
14	1981	M/16	**Yes**	—	36	high	100	married	3 children	52	cavernoma, stroke in 2014 and 2015
15	1982	M/1	**Yes**	—	35	secondary	100	single	no	36	part-time work, hearing loss
16	1983	M/2	**Yes**	—	34	secondary	100	single	no	37	meningioma, tricuspid valve insufficiency, cochlear implant
17	1983	F/7	**Yes**	—	34	secondary	100	single	2 children	41	part-time work, meningioma (resection in 2003)
18	1984	F/5	**Yes**	—	33	primary	80	single	no	37	disabled, epilepsy, meningioma (diagnosed 2010)
19	1984	F/7	**Yes**	—	33	secondary	100	single	no	41	part-time work, dementia
20	1985	F/2	**Yes**	—	32	secondary	100	single	no	34	rectal cancer, cholesteatoma, meningioma, epilepsy from 1995
21	1985	F/7	**Yes**	—	32	secondary	100	partner	no	39	part-time work, thyroid carcinoma (1997), cholesteatoma
22	1985	F/8	**Yes**	—	32	secondary	100	single	no	40	part-time work, meningioma(resection in 2003 & 2017)
23	1985	M/9	**Yes**	—	32	secondary	100	single	no	41	part-time work, head injury (1997), BCC, thyroid lesion
24	1987	F/2	**Yes**	—	30	secondary	100	single	no	32	meningioma, BCC, dementia, hearing loss

In those who died during the follow-up, the documented or supposed cause of death (whenever known) is marked in bold letters in short clinical summary.

BCC = basal cell carcinoma, BI = Barthel Index, DOD = dead of disease, DOT = dead of (late effects of) treatment. n/a = information not available

^a^age at diagnosis

^b^age at the latest follow-up

^c^this patient developed swallowing difficulties and finaly died from suffocation, after having aspirated a piece of meal and being resuscitated

^d^this patient with dementia and severe depression progressively deteriorated the last half year of her life due to heart failure; when she in addition developed a shunt failure, the family did not wish a surgical exploration of her shunt. It was respected and regarded as a palliative measure, in order to prevent the patient from further suffering.

Among multiple other complications, twelve patients (50%) developed secondary neoplasms during the follow-up, supposedly secondary to radiotherapy: meningiomas (n = 10), basal cell carcinomas (n = 3), thyroid carcinomas (n = 2) and rectal cancer (n = 1).

Three patients deceased after 22, 23 and 28 years, leading to an observed 30-year survival rate of 44%. Furthermore, another five of the long-term survivors deceased after reaching 30-year survival, most likely due to late effects of the treatment. Thus, the observed, yet incomplete 40-year survival so far is 36%.

Fourteen out of 47 patients (29.8%) were under 4 years of age at the time of diagnosis and primary treatment including radiotherapy (45–56.7 Gy, including 27–35 Gy against the whole brain). Nine of them reached 20-year survival (64.3%). From this subgroup, four patients (44.4%) died due to late effects of the treatment (Table 1) after 28, 30, 33 and 41 years of follow-up, respectively.

Barthel Index was 100 (i.e., the highest degree of independence) in all but one out of 16 long-term survivors (93.8%) and 80 in the remaining one patient who is dependent on others.

Out of 16 patients who are still alive, three (18.8%) have reached higher education, whereas twelve patients (75%) have reached secondary and one (6.3%) only primary education. Only five of these (31.3%) have been married or have had a partner, whereas eleven (68.8%) have been single. Only five patients (31.3%) have got children.

## Discussion

The present series provides long-term outcome data from a subset of patients surviving >20 years following primary treatment for posterior fossa MB in childhood. The patients included in the study were those treated after introduction of systematic postoperative radiotherapy and the first international treatment protocol in Norway in 1974, including adjuvant chemotherapy^9^, and until 1988 at which point of time it was decided that children younger than 4 years should no longer receive radiotherapy because of its detrimental late effects. Our main finding from the exceptionally long follow-up is that despite satisfactory control of tumor disease in long-term survivors during the first 20 years, they still may die from tumor recurrences even after decades of observation, but most of them from complications related to treatment.

The overall survival figures are clearly poorer than what would be expected from today’s perspective^15^, although similar as from some other reports from this period of treatment^16^. However, our data need to be regarded in the light of the historic treatment at that time, as the present series covers four decades of treatment. Beside reduced use of craniospinal radiotherapy in youngest children, there has been a significant improvement in all aspects of treatment during this period: surgical techniques and perioperative care, radiation techniques including optimizing of the standard irradiation and introduction of limited boost volume as well as proton radiotherapy, chemotherapy strategies and not least the introduction of advanced radiological technologies, particularly the MRI, in the diagnostics and surveillance of children with CNS tumors. Thus, despite being perhaps mostly of historical interest, our study reflects the lessons learned during this long period of treatment.

Among the long-term survivors, nine presented and received treatment including radiotherapy at the age under 4 years, a policy that later was abandoned (after 1988). Seven of these patients were even under 3 years of age. At the time of analysis, four of these youngest long-term survivors were deceased (44.4%) after a total follow-up of 28–41 years, while five were still alive (55.6%). As shown in Table 1, particularly these very young patients have suffered from various late complications to the treatment to the extent that barely may justify slightly more favorable effect of radiotherapy in this subgroup (20-year survival 64.3% compared to 51% for the whole cohort). However, it also appears that the morbidity related to late effects of treatment is equally significant in virtually all long-term survivors, regardless their age at the time of primary treatment.

The curative effect of postoperative radiotherapy was overwhelming in the treatment of pediatric medulloblastoma^8^. It was introduced to Norwegian patients as a routine, systematic part of their management in 1974 when they became included in the first international treatment protocol. Comparison of our own results before and during the era of radiotherapy showed clearly that half of the children with MB would be able to reach long-term survival due to radiotherapy^1^. The survival curve (Fig. 1b) then appeared to obey Collins‘ law, known as period of risk for recurrence (PRR), by which a child was most likely to become a long-term survivor if having survived for a period lasting like their own age at the diagnosis plus 9 months^17^. Follow-up data from the present study also indicates validity of this rule: the only patient who died due to late recurrence (which presented after 20 years) was almost 18 years old at the time of initial diagnosis and lived for additional two years following secondary treatment.

Patients surviving the PRR used to be regarded as cured from their primary tumor^18^ as the most recurrences appear to occur within the first 2–5 years of diagnosis^15^. Also our own data indicates excellent tumor control in the period between 5 and 20 years following primary treatment (Fig. 1), when only two patients died from tumor recurrences 5.1 and 9.9 years after the diagnosis, respectively. This fact appears even better when compared with data from the Surveillance, Epidemiology and End-Results (SEER) Program, where primary disease accounted for 59% of late deaths (while secondary malignancies accounted for 11.8%) among MB patients surviving >5 years during a mean follow-up of 16 years^19^. Therefore, it is a rather disappointing finding that one third (8/24) of our 20-years survivors died during later follow-up, although most of them probably due to consequences of their treatment. Moreover, five of these late deaths occurred after as long as 30 to 41 years following primary treatment.

Vinchon *et al*^20^. studied 207 adult survivors of pediatric brain tumors, aged 20–45 years: 23% of them presented with late progression of their initial tumor, 3.4% died of tumor progression and 14% developed new tumors requiring surgery. The main medical complications were the endocrine disturbances in 44% of patients and the cognitive in 43%; only 18% of survivors did not develop any complications at all. Here, young age at the time of irradiation was the only significant factor predicting poor employment status in the adulthood. Many other studies have documented a significant negative impact of brain tumor diagnosis in the childhood in long-term survivors^16,21–23^.

Late detrimental effects of oncological curative treatment are well known today and can be recognized in terms of late deaths as well as harmful effects on daily living and work participation^6^. As recently shown by the Finnish group, most of the negative outcomes and medical complications have a high prevalence even 10 to 30 years after primary brain tumor diagnosis^24^; in addition, the irradiated survivors had a significantly increased hazard ratio for endocrine diseases, neurological diseases, as well as visual and hearing disturbances, compared with non-irradiated survivors.

Secondary neoplasms are one of the most feared complications to radiotherapy given in childhood^15,25–27^, with cumulative 10-year incidence reported to be 4.2%^15^. In our study, the incidence was much higher (in 50% of all 20-year survivors), which may be explained by exceptionally long follow-up that we provide in this study. This is a critical point as it illustrates the time-dependent feature of secondary morbidity related to treatment in long-term survivors, and stresses the need for lifelong follow-up of these patients. Obviously, the incidence of secondary neoplasms would be only 25% when all our 47 patients with posterior fossa MB were taken in account; however, almost half of these subjects were actually not “at risk” for these late effects, as 22 out of 23 of the remaining patients died within 5.1 years after primary treatment.

In our own subgroup of long-term survivors, a number of other complications occurred: cardiovascular disease, stroke, intracerebral cavernoma formation (possessing a certain risk of spontaneous hemorrhage), progressive epilepsy as well as progressive reduction in intellectual capacity leading to dementia.

In the previous study on outcome of all children treated for MB and PNET in the same period (n = 158)^12^, we could identify severe progressive dementia as cause of death in only one patient, but incidence of dementia in whole cohort was not assessed. Among 24 long-term survivors with posterior fossa MB in the present study, there were five cases of documented dementia when the outcome was updated only four years later. It was still the same patient in whom severe progressive dementia could be clearly considered a cause of death: a highly educated lady (nr. 4 in Table 1) who was in full-time work until 30 years of follow-up but deteriorated rapidly during the next 5 years. Due to retrospective character of the present study, we did not have opportunity to investigate the patients with dementia in detail. However, the rather typical history in long-term survivors appears to be a rapid deterioration with progressive dementia and increasing dependency on others, occurring even in patients who had lived well and independently the first 20–30 years after primary treatment. This illustrates particularly well the message of our study that some late effects of treatment will clinically present first after many decades of follow-up.

Severe depression was also observed in two cases, in one patient supposedly leading to suicide.

The devastating impact of all these complications on survivors‘ quality of life is obvious and should ideally be assessed by a reliable scoring tool as well as neuropsychological tests^16,28^, as recently illustrated in another study from our group^29^; however, such investigation was beyond the scope of this particular study.

Although we undertook the histopathological review of all specimen according to WHO 2007 criteria, data from our patients could not be stratified according to MB subgroups as recognized today (SHH, WNT, Group 3 and 4). This also is a certain limitation of the present study as it is known that MB does not switch subgroup at the time of recurrence^30^. However, as there was only one late recurrence in the present cohort, such information would be of limited significance in this particular case.

By definition, long-term results should include data from children treated over a long time period before data analysis. This implies that Kaplan-Meier survival plots have many observations during the first and second decade after initial treatment and fewer observations for 30- and 40-years survival. Thus, we present 20-year *observed* survival (51%) as well as 30-year *observed* survival (44%) based on full data sets, without any patients lost to follow-up. Interestingly, the latter figure is obviously lower than that reported recently based on data from SEER^13^, where the *estimated* 30-year overall survival after treatment for MB was 70.2% (n = 876). However, in this particular study all patients surviving less than five years were excluded (620 out of 1496), and the mean follow-up was only 11.6 years. Despite this limitation, the authors address the same question as we do in our study: what happens to these children 20, 30 or 40 years after diagnosis?

In another previous study based on SEER database and evaluating cause of mortality in 5-year survivors of a central nervous system (CNS) tumors diagnosed under the age of 20 years^31^, the primary brain tumor accounted for 51% of deaths, while secondary malignancies accounted for 10% of deaths. Here, 5-year survivors of a childhood CNS tumors exhibited an almost 13-fold increased risk of death compared to their peers, and were at an increased risk of death due to both recurrent disease, secondary malignancies, as well as cerebrovascular and cardiovascular events.

While we already now may recognize that one third of our 20-years survivors are deceased as mentioned above, complete data on 40- and 50-year survival will not be available until another 10 and 20 years of further follow-up. However, at this point of time we can observe that the 40-year survival has already fallen to as low as 36%, and it will probably continue to fall until all patients from the present series reach 40 years of observation in 2027.

Taken together, the view of MB as potentially curable disease is counteracted by a life-long risk of developing complications related to the treatment given in childhood. Along with the obvious risk for tumor relapse even decades after primary treatment, this prompts the need for close, life-long follow-up of long-term survivors.

## Conclusion

Despite successful treatment of MB in childhood and satisfactory tumor control during the first 20 years following primary treatment, our data indicates that even long-term survivors may die from tumor recurrence in the following decades. However, the main factors causing late mortality and morbidity in these long-term survivors seem to be the complications related to oncological treatment given in childhood.

## References

[1] Helseth, E., Due-Tonnessen, B., Wesenberg, F., Lote, K. & Lundar, T. Posterior fossa medulloblastoma in children and young adults (0–19 years): survival and performance. *Childs Nerv Syst* 15:451–455; discussion 456 (1999) 10.1007/s003810050437 10502004

[2] Desandes E, Guissou S, Chastagner P, Lacour B (2014). Incidence and survival of children with central nervous system primitive tumors in the French National Registry of Childhood Solid Tumors. Neuro Oncol.

[3] von Hoff K (2009). Long-term outcome and clinical prognostic factors in children with medulloblastoma treated in the prospective randomised multicentre trial HIT'91. Eur J Cancer.

[4] Gudrunardottir T (2014). Treatment developments and the unfolding of the quality of life discussion in childhood medulloblastoma: a review. Childs Nerv Syst.

[5] Edelstein K (2011). Early aging in adult survivors of childhood medulloblastoma: long-term neurocognitive, functional, and physical outcomes. Neuro Oncol.

[6] Ribi K (2005). Outcome of medulloblastoma in children: long-term complications and quality of life. Neuropediatrics.

[7] Piscione PJ, Bouffet E, Mabbott DJ, Shams I, Kulkarni AV (2014). Physical functioning in pediatric survivors of childhood posterior fossa brain tumors. Neuro Oncol.

[8] Bloom HJ, Wallace EN, Henk JM (1969). The treatment and prognosis of medulloblastoma in children. A study of 82 verified cases. Am J Roentgenol Radium Ther Nucl Med.

[9] Tait DM, Thornton-Jones H, Bloom HJ, Lemerle J, Morris-Jones P (1990). Adjuvant chemotherapy for medulloblastoma: the first multi-centre control trial of the International Society of Paediatric Oncology (SIOP I). Eur J Cancer.

[10] Salloum R (2019). Late Morbidity and Mortality Among Medulloblastoma Survivors Diagnosed Across Three Decades: A Report From the Childhood Cancer Survivor Study. J Clin Oncol.

[11] Yock TI (2016). Long-term toxic effects of proton radiotherapy for paediatric medulloblastoma: a phase 2 single-arm study. Lancet Oncol.

[12] Stensvold E (2017). Outcome for children treated for medulloblastoma and supratentorial primitive neuroectodermal tumor (CNS-PNET) - a retrospective analysis spanning 40 years of treatment. Acta Oncol.

[13] Frandsen JE, Wagner A, Bollo RJ, Shrieve DC, Poppe MM (2015). Long-term life expectancy for children with ependymoma and medulloblastoma. Pediatr Blood Cancer.

[14] Mahoney FI, Barthel DW (1965). Functional evaluation: the Barthel index. Md State Med J.

[15] Packer RJ, Zhou T, Holmes E, Vezina G, Gajjar A (2013). Survival and secondary tumors in children with medulloblastoma receiving radiotherapy and adjuvant chemotherapy: results of Children’s Oncology Group trial A9961. Neuro Oncol.

[16] Frange P (2009). From childhood to adulthood: long-term outcome of medulloblastoma patients. The Institut Curie experience (1980–2000). J Neurooncol.

[17] Collins VP, Loeffler RK, Tivey H (1956). Observations on growth rates of human tumors. Am J Roentgenol Radium Ther Nucl Med.

[18] Sure U, Berghorn WJ, Bertalanffy H (1997). Collins’ law. Prediction of recurrence or cure in childhood medulloblastoma?. Clin Neurol Neurosurg.

[19] Ning MS, Perkins SM, Dewees T, Shinohara ET (2015). Evidence of high mortality in long term survivors of childhood medulloblastoma. J Neurooncol.

[20] Vinchon M, Baroncini M, Leblond P, Delestret I (2011). Morbidity and tumor-related mortality among adult survivors of pediatric brain tumors: a review. Childs Nerv Syst.

[21] Packer RJ (2003). Long-term neurologic and neurosensory sequelae in adult survivors of a childhood brain tumor: childhood cancer survivor study. J Clin Oncol.

[22] Mulhern RK, Merchant TE, Gajjar A, Reddick WE, Kun LE (2004). Late neurocognitive sequelae in survivors of brain tumours in childhood. Lancet Oncol.

[23] Macedoni-Luksic M, Jereb B, Todorovski L (2003). Long-term sequelae in children treated for brain tumors: impairments, disability, and handicap. Pediatr Hematol Oncol.

[24] Gunn ME (2015). Late morbidity in long-term survivors of childhood brain tumors: a nationwide registry-based study in Finland. Neuro Oncol.

[25] Goldstein AM, Yuen J, Tucker MA (1997). Second cancers after medulloblastoma: population-based results from the United States and Sweden. Cancer Causes Control.

[26] Olsen JH (2009). Lifelong cancer incidence in 47,697 patients treated for childhood cancer in the Nordic countries. J Natl Cancer Inst.

[27] Stavrou T (2001). Prognostic factors and secondary malignancies in childhood medulloblastoma. J Pediatr Hematol Oncol.

[28] Maddrey AM (2005). Neuropsychological performance and quality of life of 10 year survivors of childhood medulloblastoma. J Neurooncol.

[29] Stensvold, E. *et al*. Unmet rehabilitation needs in 86% of Norwegian paediatric embryonal brain tumour survivors. *Acta Paediatrica*, 10.1111/apa.15188 (2020) 10.1111/apa.15188 31977119

[30] Ramaswamy V (2013). Recurrence patterns across medulloblastoma subgroups: an integrated clinical and molecular analysis. Lancet Oncol.

[31] Perkins SM, Fei W, Mitra N, Shinohara ET (2013). Late causes of death in children treated for CNS malignancies. J Neurooncol.

